# Supplementary material for: Relative Entropy and Minimum-Variance Pricing Kernel in Asset Pricing Model Evaluation
Source: Entropy (Basel). 2020 Jun 30;22(7):721. doi: 10.3390/e22070721 (PMC7517259; doi:10.3390/e22070721)
Supplement: Supplementary file 1 [file entropy-22-00721-s001.zip › Research data/Data description.pdf]

DATA DESCRIPTION:

Using all stocks listed in the Australian Securities Exchange and macroeconomic data for Australia, the dataset comprises the following series:

1. Monthly returns for 20 size-price to cash flow portfolios, following the Fama and French (1993) methodology. (Raw data source: Datastream database)

2. Monthly returns for 25 size-book to market equity portfolios, following the Fama and French (1993) methodology. (Raw data source: Datastream database)

3. Monthly returns for 41 industry portfolios. (Raw data source: Datastream database)

4. Private final consumption expenditure, in national currency and constant prices, non-seasonally adjusted, for Australia. (Raw data source: OECD)

5. Fama and French (1993) factors (RM, SMB and HML), following the Fama and French (1993) methodology. (Raw data source: Datastream database)

6. Fama and French (2015) factors (RM, SMB, HML, RMW, and CMA), following the Fama and French (2015) methodology. (Raw data source: Datastream database)

7. Three-month interest rate of the Treasury Bill for Australia. (Raw data source: OECD)

We have produced all return series using the following data from Datastream: (i) total return index (RI series), (ii) market value (MV series), (iii) market-to-book equity (PTBV series), (iv) price-to-cash flow ratio (PC series), (v) primary SIC codes, and (vi) tax rate (WC08346 series). We use the rules suggested by Griffin, Kelly, & Nardari (2010) for excluding non-common equity securities from Datastream data.

REFERENCES:

Fama, E. F. and French, K. R. (1993). Common risk factors in the returns on stocks and bonds. *Journal of Financial Economics*, 33, 3–56.

Fama, E. F. and French, K. R. (2015). A five-factor asset pricing model. *Journal of Financial Economics*, 116, 1–22.

Griffin, J. M., Kelly, P., and Nardari, F. (2010). Do market efficiency measures yield correct inferences? A comparison of developed and emerging markets. *Review of Financial Studies*, 23, 3225–3277.
